# Substitution p.A350V in Na^+^/Mg^2+^ Exchanger SLC41A1, Potentially Associated with Parkinson's Disease, Is a Gain-of-Function Mutation

**DOI:** 10.1371/journal.pone.0071096

**Published:** 2013-08-15

**Authors:** Martin Kolisek, Gerhard Sponder, Lucia Mastrototaro, Alina Smorodchenko, Pierre Launay, Juergen Vormann, Monika Schweigel-Röntgen

**Affiliations:** 1 Institute of Veterinary-Physiology, Free University Berlin, Berlin, Germany; 2 Institute of Physiology, Pathophysiology and Biophysics, University of Veterinary Medicine, Vienna, Austria; 3 INSERM, U699, Paris, France; 4 Institute of Prevention and Nutrition, Ismaning, Germany; 5 Institute for Nutritional Physiology “Oskar Kellner”, Leibniz Institute for Farm Animal Biology, Dummerstorf, Germany; National Institutes of Health, United States of America

## Abstract

Parkinson's disease (PD) is a complex multifactorial ailment predetermined by the interplay of various environmental and genetic factors. Systemic and intracellular magnesium (Mg) deficiency has long been suspected to contribute to the development and progress of PD and other neurodegenerative diseases. However, the molecular background is unknown. Interestingly, gene *SLC41A1* located in the novel PD locus *PARK16* has recently been identified as being a Na^+^/Mg^2+^ exchanger (NME, Mg^2+^ efflux system), a key component of cellular magnesium homeostasis. Here, we demonstrate that the substitution p.A350V potentially associated with PD is a gain-of-function mutation that enhances a core function of SLC41A1, namely Na^+^-dependent Mg^2+^ efflux by 69±10% under our experimental conditions (10-minute incubation in high-Na^+^ (145 mM) and completely Mg^2+^-free medium). The increased efflux capacity is accompanied by an insensitivity of mutant NME to cAMP stimulation suggesting disturbed hormonal regulation and leads to a reduced proliferation rate in p.A350V compared with wt cells. We hypothesize that enhanced Mg^2+^-efflux conducted by SLC41A1 variant p.A350V might result, in the long-term, in chronic intracellular Mg^2+^-deficiency, a condition that is found in various brain regions of PD patients and that exacerbates processes triggering neuronal damage.

## Introduction

The crucial role of magnesium (Mg) in normal cellular physiology has been described in many reports. Thus, unsurprisingly, Mg^2+^ deficiency and/or changed intracellular Mg homeostasis (IMH) has been associated with a multitude of serious ailments among them neurodegenerative, neurological, and psychiatric disorders such as Alzheimer's disease (AD) [1], stroke [2], aggressive behavior [3], increased stress sensitivity [4], and hyperactivity [5]. In particular, several studies have suggested an association between a disturbed IMH and the incidence of Parkinson`s disease (PD) [6], [7], [8], a chronic, progressive, neurodegenerative disorder of the motor system mainly characterized by the degeneration of neurons in the *substantia nigra pars compacta* and the formation of Lewy bodies [9], [10]. PD is estimated to affect ∼1% of people over 60 years of age or ∼0.3% of the entire population in industrialized countries [11], [12]. Oyanagi and colleagues [13], [14] have shown that continuous low Mg intake for two generations induces exclusive loss of dopaminergic neurons in rats. In agreement with previous data, the results of a study conducted among the population of Guam [15] and of a more recent case control study in Sweden [6] demonstrate that low Mg intake is linked to an increased risk of idiopathic PD. By means of phosphorus magnetic resonance spectroscopy (^31^P-MRS), Barbiroli and colleagues [16] have demonstrated a significantly increased content of inorganic phosphate accompanied by a decreased concentration of free cytosolic Mg^2+^ ([Mg^2+^]_i_) in the occipital lobes of PD patients compared with healthy subjects. On the other hand, Mg has been shown to decrease negative interactions between environmental (herbicides) and molecular factors (α-synuclein) that are known to be involved in PD pathophysiology [17]. Moreover, Hashimoto and colleagues [18] have reported the significant preventive effects of Mg against 1-methyl-4-phenylpyridinium (MPP+) toxicity [19] to dopaminergic neurons.

Although these studies have demonstrated a beneficial effect of sufficient Mg intake for PD prevention and/or the deceleration of PD progression in patients, and despite an obvious link between IMH and PD pathophysiology, the underlying mechanism(s) has (have) remained elusive until now. However, the recent discovery of SLC41A1 as a Na^+^/Mg^2+^ exchanger (NME; Mg^2+^-efflux system) [20] and its localization within the newly identified PD locus *PARK16* [21], [22], [23], [24] makes this protein an interesting candidate to explain the involvement of disturbed intracellular Mg^2+^ homeostasis in PD pathophysiology.

Human *SLC41A1* has been mapped to chromosome 1q31-32 and encodes a protein consisting of 513 amino acids having a molecular mass of 56 kDa [25]. Its 5-kb transcript has been detected in most of the tested tissues in humans and mice (notably in heart, muscle, testis, thyroid gland, and kidney) [25], [26]. SLC41A1 has been characterized as an integral protein that is located in the cytoplasmic membrane [27] and that possesses 10 (strongly preferred computer-predicted model) or 11 transmembrane domains [28] with the N-terminus being oriented intracellularly [27], [28]. SLC41A1 has also been demonstrated to form hetero-oligomeric complexes. However, the identities of its binding partners and their relevance for the normal NME function of SLC41A1 *in vivo* remain uncertain [27].

The evidence for NME being involved in PD etiology has further been strengthened by the identification of PD-specific SLC41A1 variants (c.436A>G resulting in p.K146E; c.1440A>G resulting in p.P480P; and c.552+50G>A) in the Chinese population [24] and of the variant of SLC41A1 carrying the amino acid substitution p.A350V (c.1049C>T) in one PD patient of Caucasian origin [23]. Noteworthy is also the fact that *SLC41A3*, also a member of the SLC41 family, when knocked-out in mice displayed abnormal locomotor coordination (www.knockoutmouse.org; [29]).

Furthermore, the null mutation c.698G>T resulting in skipping of exon 6 of SLC41A1 (an in-frame deletion of a transmembrane helix) has been associated with a nephronophthisis-like phenotype (NPHP), therefore, suggesting that the disturbed renal Mg^2+^ homeostasis may lead to tubular defects that result in a phenotype similar to NPHP [30]. Also, SLC41A1 has been found to be over-expressed in preeclamptic placental samples with an approximately five times higher frequency than in normoevolutive placental samples [31].

In this study, we have mainly examined the effect of the substitution p.A350V potentially related to PD on the performance of the Na^+^/Mg^2+^ exchanger SLC41A1. Our findings show that the substitution p.A350V in SLC41A1 is a gain-of-function mutation leading to increased Mg^2+^ extrusion from the cell.

## Materials and Methods

### HEK293-derived cell lines/growth media and culture conditions

Tetracycline-inducible HEK293-(HA-strep-SLC41A1) and HEK293-(HA-strep-SLC41A1-p.A350V) were constructed in co-operation with Dualsystems Biotech AG. Briefly, full-length human wild-type (wt) and point-mutation-carrying *SLC41A1* cDNA was cloned into p*NTGSH* vector (Dualsystems Biotech AG) with an N-terminal HA-strep tag. Point mutation c.1049C>T (p.A350V) was introduced by PCR-site-directed mutagenesis [32]. Introduction of the mutation was confirmed by bidirectional sequencing. The obtained p*NTGSH*-*HA-strep-SLC41A1* and p*NTGSH*-*HA-strep-SLC41A1-c.1049C>T* were separately electroporated into the Flp-In™ T-REx™ HEK293 cell line (Invitrogen). Cells were placed under hygromycin selection; hygromycin-resistant clones were screened for tet-inducible expression of the wt or mutated (p.A350V) HA-strep-tagged SLC41A1. Protein expression was induced by the addition of tetracycline (1 µg.ml^−1^) for 24 hours.

HEK293-(HA-strep-SLC41A1) and HEK293-(HA-strep-SLC41A1-p.A350V) cells were cultured in Dulbecco's modified Eagle's medium (PAN Biotech) containing 10% fetal bovine serum (PAN Biotech), 4.5 g.l^−1^ glucose (Sigma-Aldrich), 2 mM glutamine (PAN Biotech), PenStrep (PAN Biotech), Normocin™ (0.1 mg.ml^−1^, Cayla), blasticidin (15 μg.ml^−1^, Cayla), and hygromycin (0.1 mg.ml^−1^, Cayla).

The preparation (cloning protocol) and culture conditions of tetracycline-inducible HEK293-(flag-SLC41A1) were as previously described [20], [27].

### Cell survival assay

HEK293 cells inducibly over-expressing wt or the p.A350V variant were grown to approximately 80% confluency, rinsed twice with PBS (PAN Biotech) and provided with fresh culture medium. Cell viability was determined, with a TC10 automated cell counter (BioRad), at 0 h and 24 h from the beginning of the induction.

### Quantitative real time PCR

To determine the transcription activity of both transgenic variants of *SLC41A1*, namely *wt* and *c.1049C>T*, by the quantitative real time PCR (q-RT-PCR) method in induced (+tet) and non-induced (-tet) cells, we used the following primers: *hSLC41A1fw*, 5′-TTGGACGCTCGCCTTGCCTG-3′ and *hSLC41A1rev*, 5′-TGGTGTGGAACACCTGCGCC-3′. Expression activities of *SLC41A2* and *SLC41A3* were determined with following primer pairs: *hSLC41A2fw*, 5′-TGGTTATAAGTAGCATTGGGGGCCT-3′ and *hSLC41A2rev*, 5′-TCCTGCTAGCCTGAATGGCCA -3′; *hSLC41A3fw*, 5′-CACAAAGATAGTCGGTATCTGACG-3′and *hSLC41A3rev*, 5′-GACCATGGCCAGGATGATT-3′. Total RNA isolation, the determination of its integrity, purity, and quantity, cDNA synthesis, and q-RT-PCR were performed as described by Kolisek and colleagues [20]. Data were evaluated with software application FK-Wolf-01, developed by Katharina Wolf (FU Berlin). Statistical evaluation was performed with data sets acquired from three biological preparations for each condition loaded in triplicate.

### Protein detection in tet-inducible HEK293 cell lines

HEK293 cells, over-expressing flag- or HA-strep-tagged wt or p.A350V, and the respective uninduced controls were lysed with RIPA buffer for 30 min. Centrifugation was performed to pellet unsolubilized material (14000 rpm, 30 min, 4°C). The total protein concentration was determined with the Bradford protein assay (Biorad). For the flag-tagged wt variant, samples containing 10 µg total protein and, for the HA-strep-tagged wild-type variant and the p.A350V mutant, 30 µg total protein were run on a 10% SDS-polyacrylamide gel and transferred to a polyvinylidene difluoride (PVDF) membrane. Immunoblotting was performed in TBS-TWEEN plus 2.5% dry milk with antibodies against the flag-tag (HRP-conjugated anti-flag M2, Sigma-Aldrich) and the strep-tag (Qiagen). Anti-mouse IgG linked to horseradish peroxidase (HRP; Cell Signaling Technology) was used as the secondary antibody for the anti-strep antibody. The antibody against RPL19 (Abnova), together with the anti-mouse secondary antibody stated above, was used to detect the loading control. Proteins were visualized by use of the SuperSignal^TM^ West Dura system (Pierce). Image J software (http://rsb.info.nih.gov/ij/) was used for the densitometric analyses.

### Membrane protein enrichment

The ProteoExtract^TM^ native membrane protein extraction kit (Calbiochem) was used to extract and enrich membrane proteins from HEK293 cell lines over-expressing wt or p.A350V according to the manufacturer's protocol. Proteins (25 µg) were separated on a 10% SDS-polyacrylamide gel. Immunoblotting was performed as previously described. The soluble protein RPL19 was used to control the specificity of the separation between soluble and membrane proteins.

### Subcellular fractionation of proteins

For fractionation of proteins according to their subcellular localization, we used the Qproteome cell compartment kit (Qiagen). Wt or p.A350V cells were induced or left untreated. Cells (4×10^6^) were processed according to the manufacturer's protocol. The obtained fractions (cytosolic, membrane, nuclear, and cytoskeletal) were electroseparated on an 8.5% SDS-polyacrylamide gel, and SLC41A1 variants were immuno-detected as described previously. As a control for the specificity of the fractionation, parallel blots were run and probed with antibodies against RPL19 (cytosolic fraction), PMCA4 (membrane fraction; Sigma-Aldrich), or Lamin A (nuclear fraction; Sigma-Aldrich). Mouse secondary antibody conjugated to HRP (Cell Signaling Technology) was used for RPL19 and PMCA4, and an HRP-coupled rabbit antibody was used for Lamin A (Cell Signaling Technology).

### Determination of the phosphorylation status of wt and p.A350V variant

The PhosphoProtein Purification Kit (Qiagen) was used according to the manufacturer's instructions. 1.5×10^7^ cells of the stably transfected HEK293 cell lines expressing strep- or flag-tagged wt or strep-tagged variant p.A350V (HA-Strep-tagged) were used as starting material. 2.5 mg of total protein was used for the affinity purification of phosphorylated proteins. Flow through (unphosphorylated proteins) and elution fractions (phosphorylated proteins) were precipitated with 8% (weight/volume) trichloroacetic acid and washed once with acetone. The pellets were dissolved in 0.1 M Tris.HCl buffer containing 2 M urea. Proteins were separated on a 10% SDS-polyacrylamide gel, transferred to a PVDF membrane and immunostained with a primary anti-strep antibody and a secondary HRP-coupled mouse antibody. For the flag-tagged cell line M2 antibody was used. To detect the phosphorylated form of Akt, the phospho-akt (Ser473) primary antibody (Cell Signaling Technology) and the secondary mouse antibody were used. Images were acquired with the BioRad ChemiDoc™ MP System (BioRad).

### Blue native electrophoresis and Western blot analysis of SLC41A1- and SLC41A1 p.A350V-protein complexes

Samples containing 10 and 20 µg of strep-affinity purified proteins (IBA & Qiagen) were loaded onto a native 5–18% polyacrylamide gradient gel, and blue native electrophoresis was performed according to Schägger and Jagow [33]. Electroseparated proteins were transferred to a PVDF membrane. Wt and p.A350V protein complexes were immuno-detected as described previously. NativeMark™ unstained protein standard (Invitrogen) was used as size marker.

### Confocal Microscopy

Specimens were prepared according to Kolisek and coworkers [27] except that, for fixation and permeabilization, we used methanol-acetone, and blocking was performed with 10% goat serum. For detection of strep-tagged wt and p.A350V, we used a primary anti-strep antibody (diluted 1∶500; Qiagen). The Alexa Fluor-647-conjugated WGA (Invitrogen) was used as a cell membrane marker. Processed samples were mounted with Fluoroshield-DAPI (abcam). Confocal images were taken with a Confocal Laser Scanning Microscope LSM 510 META (Carl Zeiss) equipped with a 63x oil-immersion objective. For the excitation of Alexa-488, Alexa-647, and DAPI, an argon-ion laser (488 nm), helium-neon laser (647 nm), and blue diode laser (405 nm) were used respectively. Image J software (http://rsb.info.nih.gov/ij/) was used for the image merging and correction, as well as to quantify the percentage of co-localization.

### Determination of free intracellular Mg^2+^ by mag-fura 2 FF-Spectrofluorometry

The -tet and +tet wt and *p.A350V* cells were rinsed twice with ice-cold, completely divalent-free, Dulbecco's phosphate-buffered saline (DPBS), detached by HyQtase, centrifuged, washed twice in completely Ca^2+^- and Mg^2+^-free Hank's balanced solution (CMF-HBS) supplemented with 10 mM HEPES and 1.36 mM L-glutamine (CMF-HBS+), and finally resuspended in the same solution. Then, the cells were loaded with 7.5 µM mag-fura 2-AM (30 min, 37°C) in the presence of the loading-facilitator Pluronic F-127 (both from Life Technologies/Molecular Probes). After being washed in CMF-HBS+, cells were incubated for a further 30 min at 37°C to allow for the complete de-esterification of the fluorescence probe, washed twice in CMF-HBS+ to remove extracellular mag-fura 2 and stored in CMF-HBS supplemented with 10 mM HEPES, 5 mM glucose, and 0.4 mM Mg^2+^ until the start of the experiments. Directly before measurements, all cells were Mg^2+^-loaded by a 20-min pre-incubation in CMF-HBS+ supplemented with 10 mM Mg^2+^ (influx conditions; [Mg^2+^]_e_ >> [Mg^2+^]_i_). Then, after the remaining extracellular Mg^2+^ had been washed out by rinses in CMF-HBS+, the [Mg^2+^]_i_ of cells was continuously determined for 10 min in CMF-HBS+ (efflux conditions in which [Mg^2+^]_i_ >> [Mg^2+^]_e_  = 0 mM; [Na^+^]_i_ << [Na^+^]_e_  = 145 mM); CMF-HBS+ supplemented with 5 mM Mg^2+^; or CMF-HBS+ supplemented with 10 mM Mg^2+^. Differentiation of NME from other transport components was performed by means of the NME inhibitor imipramine (250 µM) [20] or the NME stimulator dB-cAMP (100 µM) [20], [34].

Measurements were performed at 37°C in 3 ml cuvettes containing 2 ml cell suspension (cytocrit: 10%) while being stirred in a spectrofluorometer LS50-B (PerkinElmer) [20], [27]. [Mg^2+^]_i_ values were calculated from the 340 to 380 nm ratio according to the formula of Grynkiewicz *et al*. [35] and as described in Kolisek *et al*. [20], [27]. SLC41A1-dependent Mg^2+^ extrusion from induced SLC41A1 (wt) and p.A350V cells was determined from the [Mg^2+^]_i_ changes observed in Mg^2+^-loaded cells during recovery in CMF-HBS+ solution and calculated by subtracting the respective values of uninduced cells.

If not otherwise stated, data are presented as means ± SE. All statistical calculations were performed with Sigma-Stat (Jandel Scientific). Significance was determined by Student's *t*-test or Mann-Whitney rank sum test as appropriate; P≤0.05 was considered to be significant.

### Impedance-based measurement of cell adhesion and proliferation

The xCELLigence system (RTCA-SP, ACEA Biosciences Inc.) was used according to the manufacturer's instructions for the continuous real-time monitoring of cell adhesion and proliferation by cell-electrode impedance [36] displayed as the dimensionless Cell Index (CI). By using the RTCA Analyser, electrical impedance changes were measured across interdigitated microelectrodes integrated on the bottom of a specialised 96-well plate (E-Plate 96) and sent to the RTCA Control Subunit. The latter used the RTCA Software (version 2.0) for CI calculation from the frequency-dependent electrode resistances and real-time display of data.

Background impedance of E-Plate 96 wells was determined with 50 µl culture medium only or culture medium containing respective concentrations of tetracycline, imipramine, or dB-cAMP. Subsequently, per well, 5×10^5^ wt or p.A350V cells were plated in a final volume of 100 µl culture medium and half of the samples were induced with tetracycline. Then, localized on the RTCA SP Station the E-Plate 96 was placed into the CO_2_-incubator, and the CI was monitored every 15 minutes over a period of 48 hours. After about 24 h in culture, cells were either treated with medium or with compounds known to inhibit (imipramine, 250 µM) or activate (dB-cAMP, 100 µM) SLC41A1-dependent Mg^2+^ efflux.

## Results

### Characterization of tet-induced over-expression of SLC41A1 wt and p.A350V variants in HEK293 cells

Functional examination of both variants was performed in the newly generated HEK293 cell lines with tet-regulated expression of stably transfected HA-strep-SLC41A1 (HEK293-(HA-strep-SLC41A1); referred to further only as wt cells) and HA-strep-SLC41A1-p.A350V (HEK293-(HA-strep-SLC41A1-p.A350V); referred to further only as p.A350V cells). Thus, the basic characteristics of each cell line were acquired before the physiological characterization of the potentially PD-associated variant of SLC41A1, p.A350V, was performed.

**Figure 1:**
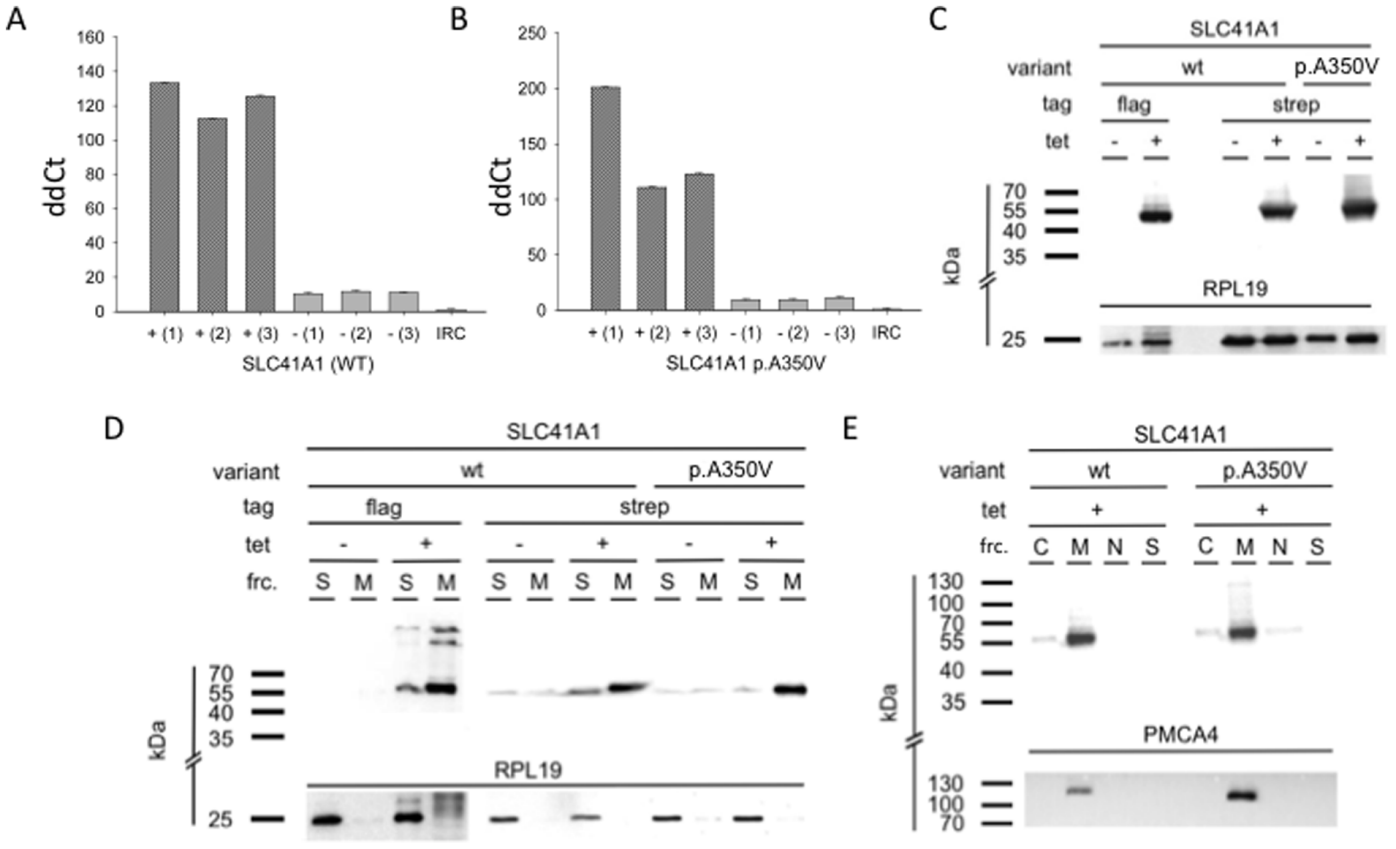
(A) Quantitative real time PCR analysis of *SLC41A1* (*wt*) expression in -tet and +tet cells. The ddCt values of three independent +tet samples and three independent -tet samples are shown. Each biological sample was loaded in triplicate. IRC indicates inter-run control. (B) Quantitative real time PCR analysis of *SLC41A1*-(*c.1049C>T*) expression in -tet and +tet cells. The ddCt values of three independent +tet samples and three independent -tet samples are given. Each biological sample was loaded in triplicate. IRC indicates inter-run control. (C) Immunodetection of recombinant HA-strep-SLC41A1(wt) and HA-strep-SLC41A1-p.A350V in total protein isolate from -tet and +tet (24 h) cells. Strep-tagged wt and p.A350V were detected only in tet-induced cells. Positive control: flag-tagged SLC41A1 isolated from HEK293 cells, clone 17, which was extensively characterized in [20], [27]. Loading was controlled by immunodetection of RPL19 protein. (D) Immunodetection of recombinant HA-strep-SLC41A1(wt) and HA-strep-SLC41A1-p.A350V in soluble and membrane-protein-enriched fractions isolated from -tet and +tet (24 h) cells. Strep-tagged wt and p.A350V were detected almost exclusively in tet-induced cells and predominantly in membrane (M) protein fractions and in much lower quantities in soluble (S) protein fractions. Positive control: flag-tagged SLC41A1 isolated from HEK293 cells (clone 17). Soluble RPL19 was used to control the specificity of the separation between soluble and membrane proteins. (E) Immunodetection of recombinant HA-strep-SLC41A1(wt) and HA-strep-SLC41A1-p.A350V in subcellular protein fractions isolated from +tet (24 h) cells. Wt and p.A350V were predominantly detected in membrane (M) protein fractions with much lower quantities in cytosolic (C) protein fractions and also for p.A350V in traces in the nuclear (N) protein fraction. Transgenic variants were not detected in cytoskeletal (S) fractions. Specificity of the fractionation was controlled on a parallel blot by immunodetection of PMCA4 (M).

Previously, we had observed that longer tet-induction (20–24 hours) of flag-SLC41A1 in HEK293 cells (clone 17) led to increased death rates of the cells [20]. Therefore, we examined whether a 24-h over-expression of SLC41A1 wt (further only wt) and SLC41A1 p.A350V (further only p.A350V) variants would be tolerated, or whether it would also result in increased death rates. After tet-induction (+tet), we determined 93% to 100% viability of the cells over-expressing either wt or p.A350V. Viability of uninduced (-tet) wt or -tet p.A350V cells ranged after 24 h between 95% and 100%, and therefore, we concluded that a 24-h tet-induced over-expression of the wt or p.A350V variant had no significant effect on cell viability. Weaker expression of wt and p.A350V was also demonstrated by the finding that we had to load a 3-fold greater amount of the protein onto the gel to be able to obtain a SLC41A1 signal equally strong as that in clone 17 (Fig. 1C) [20].

Next, we quantified the transcriptional activity of both transgenic *SLC41A1* variants after 24 h of tet-induction and in -tet cells. A significantly higher amount of ∼11.25-fold (+tet wt ddCt mean 123.72±7.42/-tet wt ddCt mean 11.0±0.48; P = .89e-09) of the wt *SLC41A1* transcript and a significantly higher amount of ∼14.25 fold (+tet c.1049C>T ddCt mean 144.9±34.62/-tet c.1049C>T ddCt mean 10.17±0.79; P = 1.84e-08) of the *SLC41A1*-c.1049C>T transcript was detected, when compared with -tet cells (Fig. 1A and 1B). Therefore, we concluded that both wt and p.A350V cells produced similar amounts of transcript after 24 h of tet-induction. We also wished to know whether the over-expression of wt *SLC41A1* or *c.1049C>T* mutant could influence the expression of *SLC41A2* and/or *SLC41A3* in +tet and -tet wt and p.A350V cells, respectively. Indeed, we did not detect any significant influence of wt or p.A350V over-expression on the expression of SLC41A2 and SLC41A3 (data not shown). This also confirmed the specificity of the *hSLC41A1fw* and *hSLC41A1rev* primers.

Leaky expression of the gene of interest can often complicate functional studies [27], [37]. Therefore, we tested whether wt and p.A350V over-expressing cell lines exhibited considerable levels of leaky expression of the wt and p.A350V variants. As a control, we used clone 17 of HEK293-(flag-SLC41A1) cells as previously characterized by Kolisek et al. [20], [27]. Western blot analysis revealed bands specific for the wt and p.A350V variants (both ∼56 kDa) almost exclusively in +tet cells (Fig. 1C). These data confirmed that both tested cell lines exhibited a negligible leaky expression of the transgenic SLC41A1 variants and, therefore, were suitable for downstream experimentation. Next, we determined whether wt and p.A350V cells expressed comparable amounts of SLC41A1 protein. With densitometric analyses performed on three blots with equal amounts of protein isolates from wt and p.A350V cells, we calculated that the density of p.A350V-specific bands was ∼1% lower compared with that of wt-specific bands. Therefore, we concluded that both wt and p.A350V cells produced nearly identical amounts of the respective SLC41A1 variants after 24 h of tet-induction. These data further underlined the suitability of both cell lines for further functional experimentation.

### SLC41A1 p.A350V localizes within the cytoplasmic membrane

**Figure 2:**
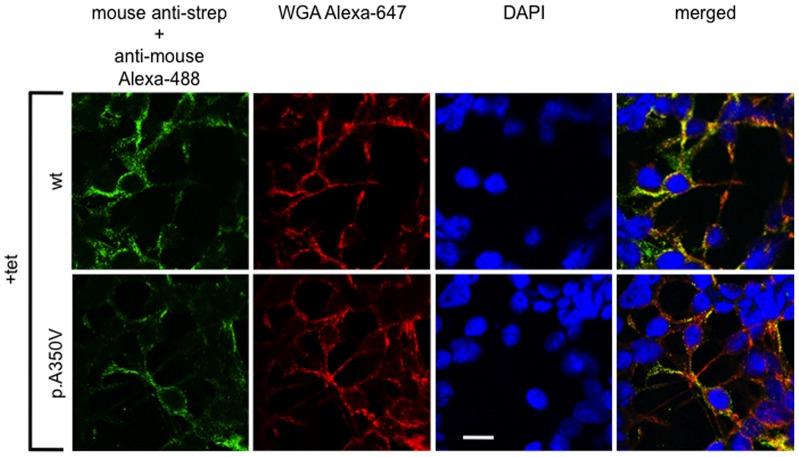
Confocal immunolocalization of HA-strep-SLC41A1 (**wt**) **and HA-strep-SLC41A1-p.A350V in +tet** (**24** **h**) **cells.** Strep-tagged wt and p.A350V were immunolabeled with primary mouse anti-strep and secondary GAM Alexa-488 antibodies (green signal). Plasma membranes were fluorescently contrasted with wheat germ agglutinin (WGA) conjugated to Alexa-647 (red signal). Nuclei were stained with DAPI (blue signal). The merged images show that both Alexa-488 and Alexa-647 signals co-localize in +tet cells. Scale bar indicates 10 μm.

Next, by performing Western blot analysis of the soluble protein- and membrane-protein-enriched cellular fractions (SF, MF) and of subcellular protein fractions (cytosolic, membrane, nuclear, and cytoskeletal), we examined whether the potentially PD-associated variant p.A350V of SLC41A1 was properly targeted into the cytoplasmic membrane, as demonstrated for wt SLC41A1 [20], [27]. Figure 1D shows a Western blot analysis of MF and SF isolated from -tet and +tet wt and p.A350V cells. The ∼56 kDa bands corresponding to wt and p.A350V were predominantly detected in the MF, with markedly lower abundance in SF of +tet cells. Flag-hSLC41A1 was used as a positive control [27]. Cytosolic protein RPL19 was used to control the specificity of the membrane fraction enrichment and, as expected, was detected only in the soluble protein fraction. We also performed subcellular protein fractionation with HEK293 cells over-expressing wt or p.A350V (Fig. 1E). Probing of the electroseparated fractions with an antibody against the strep-epitope resulted in the almost exclusive detection of both the wt and the p.A350V variants in the fraction enriched in plasma membrane proteins. The specificity of the fractionation was controlled by running and probing parallel blots with antibodies against PMCA4 (membrane fraction; positive control; Fig. 1E), against RPL19 (cytosolic fraction, data not shown), or against Lamin A (nuclear fraction; data not shown). These results were in accordance with our confocal microscopy data, which revealed that both wt and p.A350V SLC41A1 variants were predominantly localized in the plasma membrane. This was shown by co-localization of the green fluorescent signal of immunolabeled SLC41A1 variants (anti-strep:GAM-Alexa-488), with the red fluorescent signal of wheat germ agglutinin conjugated to Alexa-647 (Fig. 2). Colocalization correlation analysis between Alexa-488- and Alexa-647-specific signals in wt and p.A350V variants revealed a 94.5±1.8% (N = 6) and 92±2.3% (N = 7) overlap of the green and red pixels, respectively. In contrast, no wt- or p.A350V-specific fluorescence was seen in -tet cells ( Figure S1). Taken together, these data demonstrated the plasma membrane localization of the potentially PD-related p.A350V variant of SLC41A1. Thus, we conclude that the mutation p.A350V does not affect the intracellular localization of SLC41A1.

### p.A350V exhibits identical complex-forming abilities as SLC41A1 wt in HEK293 cells

**Figure 3:**
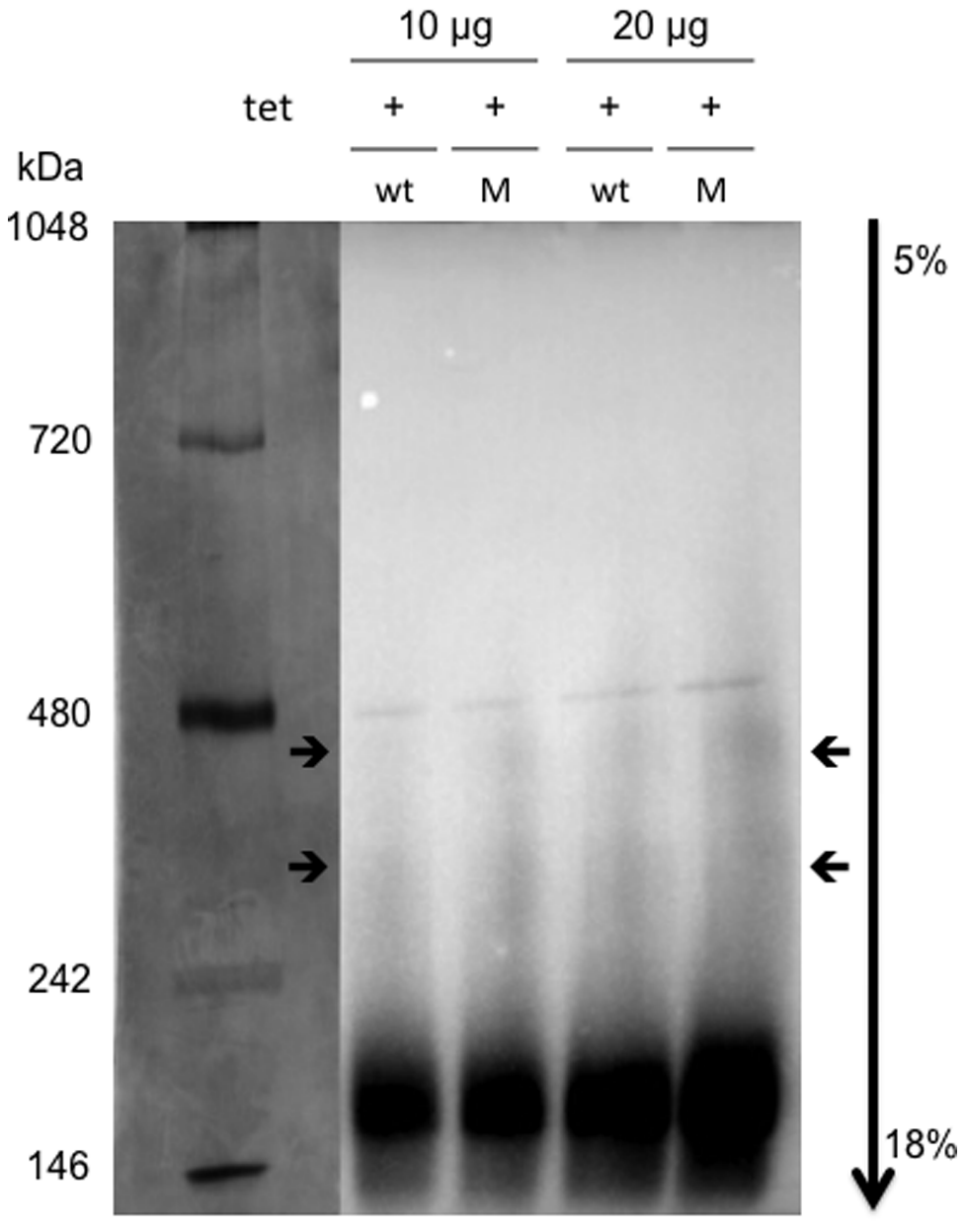
Gradient blue native PAG electroseparation (**5–18%**) **and Western blot analysis of SLC41A1**(**wt**) **and p.A350V** (**M**) **complexes.** Both wt and p.A350V variants form identical complexes (two; labeled with arrows) with molecular masses between 242 and 480 kDa.

SLC41A1 forms transient multimeric complexes *in vivo* [27]. Therefore, we wondered whether the mutation p.A350V could affect the complex-forming characteristics of SLC41A1. Figure 3 shows Western blot analysis performed on strep-affinity purified native protein isolations separated with blue native electrophoresis [27], [33]. Wt and p.A350V variants showed identical separation patterns (Fig. 3), both forming two identical complexes with molecular masses between 242 and 480 kDa. This leads us to the assumption that the mutation p.A350V has no obvious effect on the complex-forming abilities of the SLC41A1 protein.

### Amino acid substitution p.A350V in human SLC41A1 induces increased Mg^2+^ efflux

As in our previous study [20], -tet and +tet wt and p.A350V cells were Mg^2+^-loaded by a 20-min pre-incubation in solutions containing 10 mM Mg^2+^, and subsequently, the [Mg^2+^]_i_ was measured over a 10–min period in completely Mg^2+^-free solutions containing 145 mM Na^+^ (efflux conditions). [Mg^2+^]_i_ values determined at the end of each period are shown in Table 1. Uninduced wt cells and p.A350V cells regulate their [Mg^2+^]_i_ at stable levels of 0.36±0.01 mM. Compared with -tet controls, the [Mg^2+^]_i_ of +tet cells was increased by 25% (wt) and 33% (p.A350V) when incubated in 10 mM Mg^2+^ solution. After resuspension in absolutely Mg^2+^-free solutions, loaded +tet cells normalized their [Mg^2+^]_i_ to values no longer different from those of -tet cells (Table 1).

**Table 1:** [Mg^2+^]_i_ (mM) of uninduced (-tet) and induced (+tet) HEK293-(HA-strep-SLC41A1), and HEK293-(HA-strep-SLC41A1-p.A350V) cells.

[Mg^2+^]_e_ mM	HEK293-(HA-strep-SLC41A1) (control)		HEK293-(HA-strep-SLC41A1p.A350V)	
	-tet (N = 82)	+tet (N = 82)	-tet (N = 113 )	+tet (N = 113)
10	0.36±0.01	0.45±0.01**	0.36±0.01	0.48±0.01**
0	0.36±0.01	0.40±0.01^a^	0.37±0.01	0.40±0.01^a^

[Mg^2+^]_i_ values for cells successively incubated for 20 min in solutions containing 10 mM Mg^2+^ (loading conditions) and for 10 min in completely Mg-free media (efflux conditions) are given. Data are presented as means ± SE. N is being indicated. **P<0.001 vs. control (-tet cells); ^a^P<0.001 vs. loaded +tet HEK293-(HA-strep-SLC41A1) or HEK293-(HA-strep-SLC41A1-p.A350V) cells.

**Figure 4:**
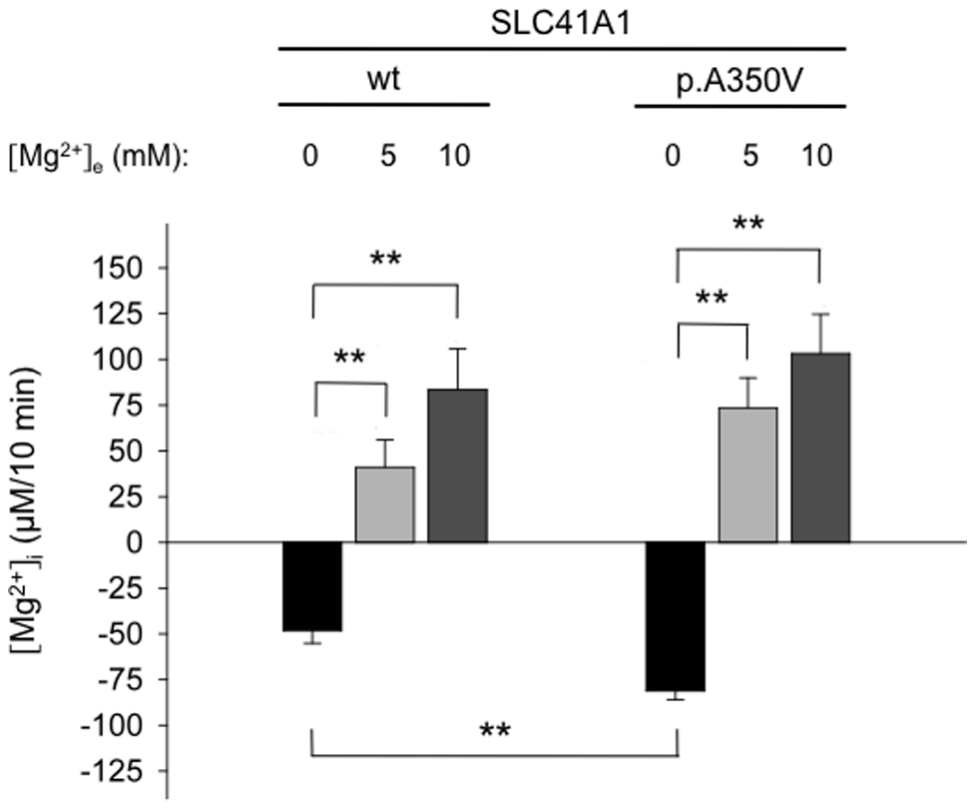
SLC41A1-related Mg^2+^ efflux in p.A350V cells compared with wt cells. Before measurements, cells were pre-loaded with Mg^2+^ as described in Material and Methods ([Mg^2+^]_e_  = 10 mM). The [Mg^2+^]_i_ change obtained after 10 min in media containing 145 mM Na^+^ is given for the following conditions: (1) completely Mg^2+^-free media (N_p.A350V_  = 113 & N_wt_  = 82); (2) media supplemented with 5 mM Mg^2+^ (N_p.A350V_  = 13 & N_wt_  = 14); and (3) media supplemented with 10 mM Mg^2+^ (N_p.A350V_  = 13 & N_wt_  = 14). Values have been corrected for [Mg^2+^]_i_ changes in -tet cells and are given as means ± SE; **P<0.001.

The [Mg^2+^]_i_ decrease observed in +tet cells was previously shown to reflect the SLC41A1-dependent Mg^2+^ efflux [20]. The results for +tet wt and +tet p.A350V cells are summarized in figure 4 showing a significantly stronger Mg^2+^ extrusion of 81.2±4.7 µM/10 min (Np._A350V_  = 113) in p.A350V cells compared with 48.2±7.0 µM/10 min (N_wt_  = 82) in the wt cells used as control. These data clearly demonstrate an increased efflux capacity of p.A350V cells.

However, if the measurements were performed in solutions containing 5 or 10 mM Mg^2+^, thereby lowering the inside-out Mg^2+^ gradient, no Mg^2+^ extrusion occurred from Mg^2+^-loaded +tet wt and +tet p.A350V cells. Instead, as can be seen in figure 4, the [Mg^2+^]_i_ increased by 41.1±15.0 µM/10 min and 83.5±22.3 µM/10 min in wt cells (Nwt; _[Mg_^2+^_]e (5 mM)_  = 14 & Nwt; _[Mg_^2+^_]e (10 mM)_  = 14) and by 73.6±16.4 µM/10 min and 103.2±21.5 µM/10 min in p.A350V cells (N_p.A350V; [Mg_^2+^_]e (5 mM)_  = 13 & N_p.A350V; [Mg_^2+^_]e (10 mM)_  = 13) incubated in 5 or 10 mM Mg^2+^, respectively, during the measurements.

### Effects of imipramine and of dB-cAMP on SLC41A1-related Mg^2+^ efflux from +tet wt and p.A350V cells

**Figure 5:**
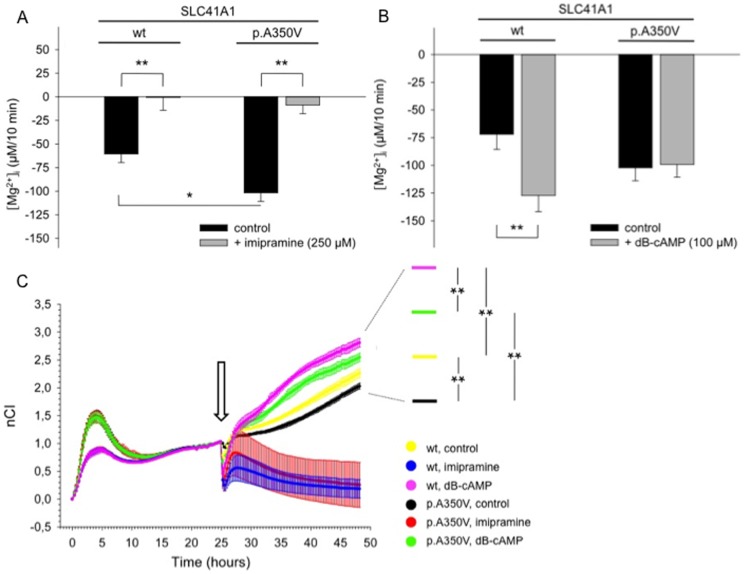
Effect of imipramine and of cAMP-dependent PKA phosphorylation on SLC41A1-related [Mg^2+^]_i_ changes, cell adhesion, and cell proliferation in +tet p.A350V cells and wt cells. A: Summary of [Mg^2+^]_i_ changes after resuspension of Mg^2+^-loaded +tet p.A350V cells and wt cells in completely Mg^2+^-free Na^+^-containing solutions with or without (control) the Na^+^/Mg^2+^ exchanger inhibitor imipramine (250 µM). Values have been corrected for [Mg^2+^]_i_ changes in -tet cells and given as means ± SE; N_p.A350V_  = 26 & N_wt_  = 21 single experiments per condition; *P = 0.03; **P<0.005. B: Summary of [Mg^2+^]_i_ changes after resuspension of Mg^2+^-loaded +tet p.A350V cells and wt cells in completely Mg^2+^-free Na^+^-containing media with or without (control) the Na^+^/Mg^2+^ exchanger activator dB-cAMP (100 µM). Values have been corrected for [Mg^2+^]_i_ changes in -tet cells and are means ± SE; Np.A350V  = 15 & Nwt  = 15 single experiments per condition; **P = 0.01. C: Original growth curves of +tet p.A350V cells and wt cells under control conditions and after application of 250 µM imipramine and of 100 µM dB-cAMP. Cells were seeded at a density of 10×10^5^ per well, induced with tetracycline, and allowed to attach and proliferate for 24 h prior to treatment with the compounds (indicated by the arrow). The Cell Index, a dimensionless parameter reflecting cell adherence and number, was normalized (nCI) to the time just before modulator application. Values are means ± SD; N = 6 single experiments per condition; **P<0.001.

In both wt- and p.A350V-over-expressing cells, the observed [Mg^2+^]_i_ decrease (−60.5±9.2 µM/10 min, N_wt_  = 21; and 101.7±9.2 µM/10 min, N_p.A350V_  = 26) was nearly completely abolished (−0.7±13.5 µM/10 min, N_wt_  = 21; and 8.7±9.1 µM/10 min, N_p.A350V_  = 26) by the tricyclic antidepressant imipramine (Fig. 5A) known to inhibit the NME function of SLC41A1 [20].

**Figure 6:**
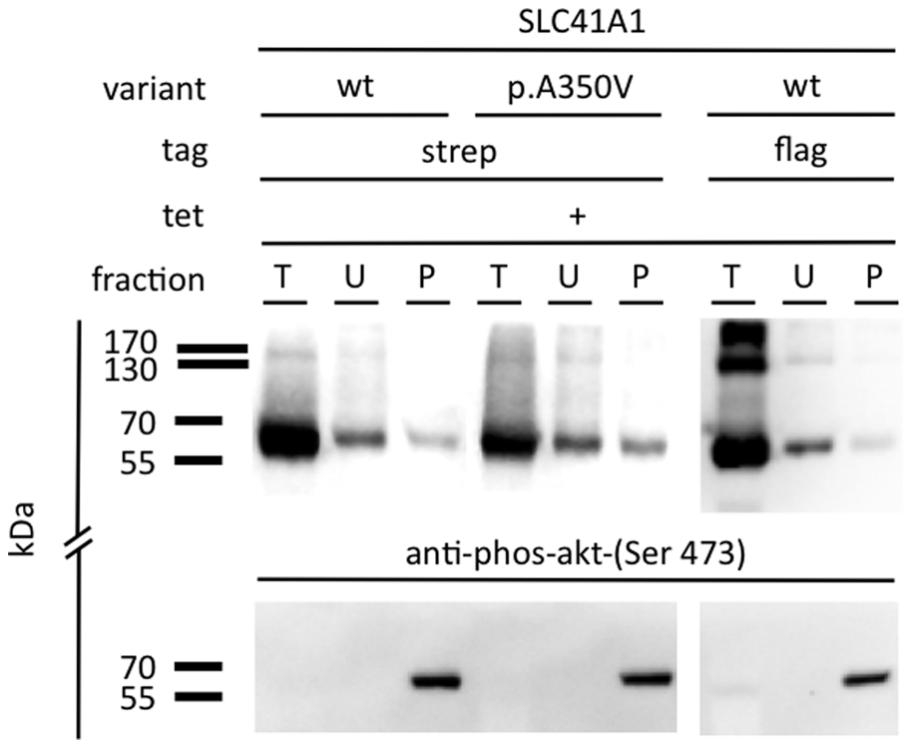
Immunodetection of phosphorylated recombinant flag-SLC41A1 (**wt**)**, HA-strep-SLC41A1** (**wt**)**, and HA-strep-SLC41A1-p.A350V with the PhosphoProtein purification kit** (**Qiagen**)**.** Total (T), flow-through (U; containing unphosphorylated proteins) and elution (P; containing phosphorylated proteins) fractions were probed with antibodies against strep- or flag-tag. A signal specific for phosphorylated wt or p.A350V was detected in all three cell lines. The specificity of the fractionation was controlled with an antibody against phosphorylated Akt.

In our previous study [20], we have demonstrated that phosphorylation, postulated to be a mechanism for the activation of Mg^2+^ extrusion [20], [34], regulates NME activity of SLC41A1 and that wt is being detectable in the phosphoprotein-specific fraction (P). To this end we performed Western blot analysis on fractionated protein lysates of induced +tet flag-wt, strep-wt, and strep-p.A350V. Figure 6 demonstrates that the ∼56 kDa bands corresponding to wt and p.A350V were detected in the P fractions. The specificity of the fractionation was controlled with an antibody exclusively recognizing phosphorylated Akt.

We also tested whether the SLC41A1-dependent Mg^2+^ efflux from +tet wt and p.A350V cells could be further stimulated by the application of dibutyryl-cAMP (dB-cAMP), a membrane-permeant cAMP analog that activates the holoenzyme complex of protein kinase A (PKA) [20]. No additional effects on Mg^2+^ extrusion from p.A350V cells were observed after the application of 100 µM dB-cAMP (N_p.A350V_  = 15; Fig. 5B). However, as shown in figure 5B, dB-cAMP increased Mg^2+^ release by 77±20% (N_wt_  = 15) in wt controls.

### Amino acid substitution p.A350V in human SLC41A1 effects cell growth

The 48-h growth curves of +tet p.A350V cells and wt cells obtained under control conditions and with imipramine or dB-cAMP in the culture medium are displayed in figure 5C. Compared with +tet wt cells, the normalized CI (nCI) of +tet p.A350V cells was reduced (2.03±0.03 vs. 2.26±0.08; P<0.001) after 48 h in culture (Fig. 5C). Application (24 h after seeding) of imipramine and of dB-cAMP in parallel to its effects on NME activity decreases and increases the 48-h nCI of +tet wt and p.A350V cells compared with control values (Fig. 5C). After imipramine application, the 48-h nCI amounted to 0.18±0.17 in wt and to 0.26±0.40 in p.A350V cells, thus showing a strong reduction in both groups. However, the dB-cAMP-induced increase of the 48-h nCI was much stronger in +tet wt cells compared with +tet p.A350V cells (2.81±0.08 vs. 2.55±0.08; P<0.001).

## Discussion

Most PD cases are sporadic with unclear multifactorial etiologies (idiopathic PD). Only approximately 3% to 5% among all sporadic PD cases are attributable to defects in seven PD-associated genes: *SNCA* (autosomal dominant pattern of inheritance; (ADPI)), *LRRK2* (ADPI), *EIF4G1* (ADPI), *VPS35* (ADPI), *parkin/PARK2* (autosomal recessive pattern of inheritance; (ARPI)), *DJ-1/PARK7* (ARPI), and *PINK1* (ARPI) [38], [39]. However, changes in several other genes have been suggested as causes for recessive neurological/neurodegenerative disorders that may include PD: hereditary ataxias (*ATXN2/3, FMR1*), frontotemporal dementia (e.g. *MAPT*) and others (e.g. *ATP13A2, PLA2G6, FBXO7*) [39].

*RAB7L1*, and *SLC41A1* have been identified within the newly revealed PD-susceptibility locus *PARK16* at chromosome 1q32 [21], [22], [23], [24]. SLC41A1 has been shown by our group to be a cell-membrane-localized Mg^2+^ carrier, conducting the exchange of intracellular Mg^2+^ for extracellular Na^+^ (NME) [20], [27]. NME has been shown to be functionally active in nearly all investigated cells and tissues including neurons [34], [40], [41], [42], [43]. It has also been shown to be responsible for the maintenance of an optimal [Mg^2+^]_i_ for a variety of processes critical for the cell such as bioenergetics [44], the regulation of cellular pH, volume, and the total ion balance, [45], [46], and cell proliferation and differentiation [47], [48].

Recently, in one PD patient, Tucci and colleagues [23] have found a coding variant of SLC41A1, carrying substitution p.A350V. Here, we have investigated if this potentially PD-associated mutation affects the molecular and/or functional properties of SLC41A1. Our experiments have revealed no changes regarding the cellular localization, phosphorylation status, or complex-forming ability of the p.A350V variant when compared with the wt protein. However, we have demonstrated that +tet p.A350V cells are able to perform Mg^2+^-efflux more efficiently than +tet wt cells. Under our experimental conditions, short-term, 10-min Mg^2+^ release is increased by 69±10% (P<0.001) after the induction of p.A350V over-expression compared with cells over-expressing wt. Moreover, as a consequence of an enhanced NME activity, we found a reduced proliferation rate in p.A350V compared to wt cells. As the growth experiments were performed for long periods (48 h) and with cells incubated in complete culture media containing 1.2 mM Mg^2+^, the effects of the p.A350V mutation seem to be of relevance also under physiological conditions.

In both cell lines, Mg^2+^ extrusion is blocked by >90% after imipramine application, clearly showing that it results from SLC41A1-mediated NME activity [42]. Imipramine, which besides quinidine, is the current pharmacological choice for NME inhibition [20], [49], is known to act on the extracellular Na^+^-binding site of the NME, and competition between these two compounds slows Mg^2+^ efflux. In agreement with this, the inhibitory effect of imipramine is in the order of that of sodium withdrawal, which amounts to 91% in our previous study with SLC41A1-over-expressing HEK293 cells [20].

Another characteristic feature of NME is its activation by PKA-dependent phosphorylation [20], [34]. Elevation of the intracellular cAMP concentration specifically stimulates Na^+^-dependent efflux either directly via an increased affinity of the transporter for intracellular Mg^2+^ [34], [50] or by Mg^2+^ mobilization from intracellular organelles, e.g. from mitochondria [51] **.** Interestingly, the application of dB-cAMP (a cell-membrane-permeant cAMP-analog) increased (77±20%) Mg^2+^ efflux only in +tet wt cells, whereas no effect has been seen in p.A350V cells. This insensitivity of mutant NME to cAMP stimulation might be of pathophysiological importance as under normal conditions various hormones or mediators, e.g., adrenergic substances, prostaglandin E2, and angiotensin II, use this pathway to induce a transient [Mg^2+^]_i_ decrease that directly or indirectly influences cellular transport mechanisms and physiological functions [49], [51], [52]. For example, in this study, the growth-promoting effect of dB-cAMP is reduced in p.A350V-over-expressing cells compared with wt cells. The inability further to increase p.A350V-related NME function via cAMP also suggests maximum or near-maximum activation of the transporter and is in accordance with the observation of enhanced Mg^2+^ efflux in mutants compared with wt cells. The [Mg^2+^]_i_, a main determinant of NME activity [53], is similar (0.44±0.01 mM) between wt and p.A350V cells. Therefore, one can speculate that the p.A350V mutation augments the affinity of the transport protein for intracellular Mg^2+^, changes the Mg^2+^-carrier-complex formation, or dysregulates transporter gating to facilitate the Mg^2+^ transport rate.

In the long-term, the increased activity of the NME might contribute to the development of intracellular Mg^2+^ deficiency [43], if not compensated for by Mg^2+^ influx. Mg deprivation, whether by gene defects such as p.A350, toxins (rotenone, MPTP), or restricted Mg intake, induces and/or exacerbates processes such as oxidative stress accompanied by an increase in NO and free radicals [54], [55], dysfunction of mitochondria and the endoplasmic reticulum [56], [57], impairment of Ca^2+^ homeostasis [58], iron accumulation [59], alterations in the autophagy-lysosome pathways, protein mishandling, and inflammatory responses [58], all of which are known to trigger neuronal damage in neurodegenerative diseases including PD [56], [60], [61]. In accordance, Oyanagi and colleagues [14] have been able to induce the severe loss of dopaminergic neurons in rats fed for one year with an Mg-restricted diet containing only one-fifth of the normal Mg content. Furthermore, a lower concentration of Mg in various brain regions and in the cerebrospinal fluid of PD patients has been found [16], [62], [63]. On the other hand, a high extracellular [Mg] of ≥1.2 mM has been demonstrated to protect dopaminergic neurons of the *substantia nigra* from MPP+ toxicity [18] and, because of its Ca^2+^-antagonizing effects, to reduce neuroinflammation [64]. Moreover, spontaneous and Fe^2+^-induced accelerated aggregation of α-synuclein can be inhibited by 0.8 mM Mg^2+^ [65]. In this study, by using an extracellular [Mg] of 5 and 10 mM, we have been able to block SLC41A1-related Mg^2+^ efflux in both wt and p.A350V cells. Increasing the extracellular Mg^2+^ concentration will reduce the driving force for an electroneutral Mg^2+^ efflux and suggests that the exchanger switches to the reverse mode, thereby performing Mg^2+^ uptake [27], [42]. Thus, Mg supplementation might be useful for preventing a loss of intracellular Mg^2+^, a loss that is detrimental to neurons.

Under physiological conditions, an adequate Mg intake should be seen as an important positive environmental factor protecting neurons against accelerated ageing caused by slowly acting deleterious environmental factors (e.g. toxins) and/or genetic risk factors. With regard to the latter, Mg^2+^ is an essential co-factor in almost all enzymatic systems involved in DNA processing and in nucleotide excision repair, base excision repair, and mismatch repair [66]. Furthermore, PD-relevant genes with a recessive pattern of inheritance (*parkin/PARK2*, *PINK1*, and *DJ-1/PARK7*) are all related to mitochondria dysfunction and oxidative stress making it possible that defects in these genes and disturbances of IMH intervene in these pathways to induce nigral mitochondrial cytopathy [67].

## Conclusions

Magnesium deficiency (both systemic and intracellular) has long been suspected to be involved in various human disease complexes such as metabolic syndrome and neurodegeneration in general including PD. An obvious molecular link between disturbed IMH and PD is however missing. In this study, we have examined the functional properties of a recently identified potentially PD-associated coding variant of the NME SLC41A1, p.A350V. We have demonstrated that p.A350V is able to perform Mg^2+^-extrusion more efficiently than wt NME and shows insensitivity to cAMP stimulation and have found a reduced proliferation rate in p.A350V compared with wt cells. Our data therefore indicate that the rare conservative substitution p.A350V is a gain-of-function mutation leading to an increased Mg^2+^ efflux capacity with likely a long-term consequence in systemic deterioration, particularly under conditions of low extracellular Mg^2+^ concentration. By extrapolation, our data are in agreement with the findings of epidemiological and case-control studies and suggest that the chronic loss of Mg^2+^ from brain tissue and, thus, latent intracellular hypomagnesemia, contributes to neurodegeneration. Na^+^/Mg^2+^ exchange in neurons and SLC41A1 *per se* might therefore represent a PD-relevant therapeutic target, with Mg^2+^ supplementation of PD patients possibly being beneficial.

## Supporting Information

Figure S1 Confocal immunolocalization of HA-strep-SLC41A1 (wt) and HA-strep-SLC41A1-p.A350V in -tet (24 h) cells. Strep-tagged wt and p.A350V were immunolabeled with primary mouse anti-strep and secondary GAM Alexa-488 antibodies (green signal). Plasma membranes were fluorescently contrasted with wheat germ agglutinin (WGA) conjugated to Alexa-647 (red signal). Nuclei were stained with DAPI (blue signal). Scale bar indicates 10 μm.
(DOC)
