# Supplementary figures and images for: Substitution p.A350V in Na+/Mg2+ Exchanger SLC41A1, Potentially Associated with Parkinson's Disease, Is a Gain-of-Function Mutation
Source: PLoS One. 2013 Aug 15;8(8):e71096. doi: 10.1371/journal.pone.0071096 (PMC3744568; doi:10.1371/journal.pone.0071096)

**Supporting figure S1**

**
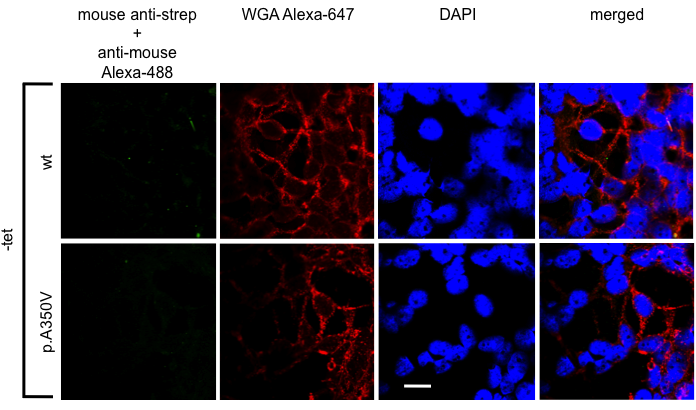
**

Supplement: Figure S1 — Confocal immunolocalization of HA-strep-SLC41A1 (wt) and HA-strep-SLC41A1-p.A350V in -tet (24 h) cells. Strep-tagged wt and p.A350V were immunolabeled with primary mouse anti-strep and secondary GAM Alexa-488 antibodies (green signal). Plasma membranes were fluorescently contrasted with wheat germ agglutinin (WGA) conjugated to Alexa-647 (red signal). Nuclei were stained with DAPI (blue signal). Scale bar indicates 10 μm. (DOC) [file pone.0071096.s001.doc]
